# Update on Vaccine-Derived Poliovirus Outbreaks — Worldwide, January 2021–December 2022

**DOI:** 10.15585/mmwr.mm7214a3

**Published:** 2023-04-07

**Authors:** John Paul Bigouette, Elizabeth Henderson, Mohamed A. Traoré, Steven G. F. Wassilak, Jaume Jorba, Frank Mahoney, Omotayo Bolu, Ousmane M. Diop, Cara C. Burns

**Affiliations:** ^1^Global Immunization Division, Center for Global Health, CDC; ^2^Division of Viral Diseases, National Center for Immunization and Respiratory Diseases, CDC; ^3^Polio Eradication Department, World Health Organization, Geneva, Switzerland.

Circulating vaccine-derived poliovirus (cVDPV) outbreaks* can occur when oral poliovirus vaccine (OPV, containing one or more Sabin-strain serotypes 1, 2, and 3) strains undergo prolonged circulation in under-vaccinated populations, resulting in genetically reverted neurovirulent virus (*1**,**2*). Following declaration of the eradication of wild poliovirus type 2 in 2015 and the global synchronized switch from trivalent OPV (tOPV, containing Sabin-strain types 1, 2, and 3) to bivalent OPV (bOPV, containing types 1 and 3 only) for routine immunization activities^†^ in April 2016 (*3*), cVDPV type 2 (cVDPV2) outbreaks have been reported worldwide (*4*). During 2016–2020, immunization responses to cVDPV2 outbreaks required use of Sabin-strain monovalent OPV2, but new VDPV2 emergences could occur if campaigns did not reach a sufficiently high proportion of children. Novel oral poliovirus vaccine type 2 (nOPV2), a more genetically stable vaccine than Sabin OPV2, was developed to address the risk for reversion to neurovirulence and became available in 2021. Because of the predominant use of nOPV2 during the reporting period, supply replenishment has frequently been insufficient for prompt response campaigns (*5*). This report describes global cVDPV outbreaks during January 2021–December 2022 (as of February 14, 2023) and updates previous reports (*4*). During 2021–2022, there were 88 active cVDPV outbreaks, including 76 (86%) caused by cVDPV2. cVDPV outbreaks affected 46 countries, 17 (37%) of which reported their first post-switch cVDPV2 outbreak. The total number of paralytic cVDPV cases during 2020–2022 decreased by 36%, from 1,117 to 715; however, the proportion of all cVDPV cases that were caused by cVDPV type 1 (cVDPV1) increased from 3% in 2020 to 18% in 2022, including the occurrence of cocirculating cVDPV1 and cVDPV2 outbreaks in two countries. The increased proportion of cVDPV1 cases follows a substantial decrease in global routine immunization coverage and suspension of preventive immunization campaigns during the COVID-19 pandemic (2020–2022) (*6*); outbreak responses in some countries were also suboptimal. Improving routine immunization coverage, strengthening poliovirus surveillance, and conducting timely and high-quality supplementary immunization activities (SIAs) in response to cVDPV outbreaks are needed to interrupt cVDPV transmission and reach the goal of no cVDPV isolations in 2024.

## cVDPV Outbreaks

Poliovirus outbreaks are considered interrupted by the World Health Organization (WHO) International Health Regulations Emergency Committee on International Poliovirus Transmission when ≥13 months have passed since the onset of paralysis in the latest case or isolation sample date (*4*). A total of 172 cVDPV outbreaks have been reported since 2016, 88 (51%) of which were active during 2021–2022 (Table 1). Among these, transmission was interrupted in 38 (42%) active outbreaks (Supplementary Table, https://stacks.cdc.gov/view/cdc/126383). This report does not describe 84 (49%) cVDPV outbreaks in which transmission was interrupted before 2021.

**TABLE 1:** Ongoing circulating vaccine-derived poliovirus outbreaks detected (N = 50), by serotype, emergence group, detection source, and other selected characteristics — worldwide, January 2021–December 2022

WHO Region	Country	cVDPV emergence designation*	Years detected	No. of detections (source)^†^			% VP1 genome region divergence from Sabin-strain poliovirus^¶^	Outbreak confirmation date	Most recent case/positive specimen from healthy child/ environmental sample**
				AFP cases	Other human sources (non-AFP)^§^	ES			
**cVDPV type 1 outbreaks**									
AFR	DRC	RDC-TAN-1	2022	88	4	0	1–2	Sep 12, 2022	Dec 16, 2022
		RDC-HLO-3	2022	3	0	0	2	Nov 14, 2022	Sep 30, 2022
	Madagascar	MAD-SUE-1	2020–22	15	22	78	3–5	Apr 26, 2021	Oct 26, 2022
		MAD-ANO-2	2021–22	8	8	87	4–6	Feb 28, 2022	Oct 25, 2022
		MAD-ANO-1	2021–22	3	2	16	1–2	Aug 2, 2021	Apr 25, 2022
	Malawi	MOZ-NPL-2	2022	4	1	0	5–6	Sep 19, 2022	Dec 1, 2022
	Mozambique	MOZ-NPL-2	2020–22	18	1	0	5	Jul 25, 2022	Nov 20, 2022
**cVDPV type 2 outbreaks**									
AFR	Algeria	NIE-ZAS-1	2022	3	2	44	3–4	Jul 11, 2022	Dec 27, 2022
	Benin	NIE-ZAS-1	2022	11	1	8	3–5	Jun 27, 2022	Dec 21, 2022
	Botswana	RDC-MAN-5	2022	0	0	6	2	Oct 31, 2022	Dec 13, 2022
	Burkina Faso	NIE-JIS-1	2019–21	2	0	1	4–5	Jan 27, 2020	Dec 28, 2021
	Cameroon	NIE-ZAS-1	2021–22	5	3	1	3–4	Oct 25, 2021	Oct 30, 2022
	Central African Republic	NIE-ZAS-1	2021–22	1	0	2	3–4	Nov 29, 2021	Dec 26, 2022
		CAF-BNG-2	2022	3	0	8	1–2	Aug 22, 2022	Nov 23, 2022
	Chad	NIE-ZAS-1	2021–22	44	3	7	3–5	Jan 31, 2022	Nov 24, 2022
	Côte d’Ivoire	NIE-ZAS-1	2022	0	0	4	2–3	Mar 7, 2022	Jul 18, 2022
	DRC	RDC-MAN-3	2021–22	253	13	5	1–3	Dec 20, 2021	Dec 10, 2022
		RDC-MAN-5	2021–22	21	4	5	1–3	Mar 14, 2022	Nov 21, 2022
		RDC-BUE-1	2022	5	10	0	2–4	Sep 5, 2022	Nov 10, 2022
		RDC-MAN-4	2021–22	11	2	4	1–2	Jan 31, 2022	Sep 20, 2022
		RDC-TSH-1	2022	4	0	0	2	Oct 3, 2022	Sep 20, 2022
		RDC-MAN-2	2021–22	5	3	3	1–2	Nov 1, 2021	Jul 5, 2022
	Eritrea	CHA-NDJ-1	2021–22	2	0	0	3–4	Jun 6, 2022	Mar 2, 2022
	Ethiopia	ETH-SOU-3	2020–22	1	0	0	3	Nov 21, 2022	Apr 1, 2022
	Ghana	NIE-ZAS-1	2022	3	7	37	4–5	May 23, 2022	Oct 4, 2022
	Mauritania	NIE-JIS-1	2021	0	4	9	4–5	Aug 23, 2021	Dec 15, 2021
	Mozambique	MOZ-NPL-1	2021–22	6	0	0	2–4	Feb 14, 2022	Mar 26, 2022
	Niger	NIE-ZAS-1	2021–22	29	3	16	2–5	Nov 1, 2021	Oct 27, 2022
	Nigeria	NIE-ZAS-1	2020–22	413	222	670	2–6	Sep 18, 2020	Dec 14, 2022
		NIE-SOS-7	2019–22	30	12	16	2–4	May 2, 2020	Jan 29, 2022
	Senegal	NIE-JIS-1	2020–22	17	36	26	4–6	Mar 16, 2021	Jan 17, 2022
	Togo	NIE-ZAS-1	2022	2	0	1	4–5	May 16, 2022	Sep 30, 2022
		NIE-JIS-1	2019–22	0	0	1	4	Oct 17, 2019	Mar 22, 2022
	Zambia	RDC-MAN-5	2022	0	0	3	2	Nov 7, 2022	Nov 1, 2022
AMR	United States	IUUC-2022	2022	1	0	12	1	Sep 12, 2022	Sep 22, 2022
EMR	Djibouti	YEM-TAI-1	2021–22	0	0	29	1–2	Jan 31, 2022	May 22, 2022
	Egypt	NIE-ZAS-1	2022	0	0	2	3	Jun 6, 2022	Aug 29, 2022
		YEM-TAI-1	2021–22	0	0	3	2	Dec 20, 2021	Mar 30, 2022
		EGY-QEN-1	2021–22	0	0	3	1–2	Mar 28, 2022	Mar 9, 2022
	Somalia	SOM-BAN-1	2017–22	6	4	4	7–9	Feb 12, 2018	Aug 31, 2022
		YEM-TAI-1	2022	0	0	1	1	Aug 22, 2022	May 19, 2022
	Sudan	NIE-ZAS-1	2022	1	0	1	4–5	Dec 19, 2022	Nov 28, 2022
	Yemen	YEM-TAI-1	2021–22	219	51	70	1–3	Nov 22, 2021	Dec 2, 2022
		YEM-SAN-1	2021–22	6	2	1	1–2	Apr 18, 2022	Aug 17, 2022
EUR	Israel	IUUC-2022	2022	0	0	1	—^††^	Aug 8, 2022	Jun 16, 2022
	UK	IUUC-2022	2022	0	0	5	—^††^	Sep 5, 2022	Aug 8, 2022
	Ukraine	PAK-GB-1	2021	2	18	0	—^††^	Oct 11, 2021	Dec 24, 2021
SEAR	Indonesia	INO-ACE-1	2022	1	4	0	3	Nov 28, 2022	Nov 11, 2022
**cVDPV type 3 outbreaks**									
EMR	Palestinian territories	cVDPV3	2021–22	0	0	16	—^††^	Mar 7, 2022	Mar 12, 2022
EUR	Israel	cVDPV3-ISR	2020–22	1	3	31	—^††^	Dec 13, 2021	Mar 24, 2022

**Abbreviations:** AFP = acute flaccid paralysis; AFR = African Region; AMR = Region of the Americas; cVDPV = circulating vaccine-derived poliovirus; DRC = Democratic Republic of the Congo; EMR = Eastern Mediterranean Region; ES = environmental surveillance; EUR = European Region; OPV = oral poliovirus vaccine; SEAR = South-East Asian Region; UK = United Kingdom; VDPV = vaccine-derived poliovirus; VP1 = poliovirus capsid viral protein 1; WHO = World Health Organization.

* Emergences indicate detection of cVDPV strains that have unique genetic reversion compared with other VDPVs, and the names of emergences generally designate the country and geographic subnational region of the emergence’s first detection and the number of emergences in each subnational region. The emergence designation for cVPDV2 outbreaks in Israel, UK, and United States is the same (IUUC-2022), because of shared circulation in each of a unique Sabin-like virus.

^†^ During January 2021–December 2022 with data as of February 14, 2023. For AFP cases, the number with a VDPV-positive specimen or for which a direct contact of the patient had a VDPV-positive specimen when the patient did not. For other human sources, the number of contacts of the patient or healthy children in the community with a VDPV-positive specimen. For detections from ES, the total number of samples with VDPVs detected from environmental (sewage) collections.

^§^ Specimens from contacts of polio patients and from healthy children in the community during January 2021–December 2022.

^¶^ Percentage of divergence is estimated from the number of nucleotide differences in the genome region encoding VP1 from the corresponding parental Sabin strain.

** For AFP cases, dates refer to the date of paralysis onset. For contacts, healthy children, and environmental (sewage) samples, dates refer to the date of collection during January 2021–December 2022 with data current as of February 14, 2023. Table is restricted to outbreaks with a last reported detection after November 1, 2021 (indicating country virus circulation within 13 months of the reporting period).

^††^ Data not released or available.

## cVDPV1 Outbreaks

Since 2016, 14 cVDPV1 outbreaks from 12 emergences^§^ have been reported across 10 countries. Nine of these 14 outbreaks were active in five countries (Democratic Republic of the Congo [DRC], Madagascar, Malawi, Mozambique, and Yemen) during 2021–2022, including five new cVDPV1 outbreaks detected in four countries (DRC, Madagascar, Malawi, and Mozambique) (Table 1) (Supplementary Table, https://stacks.cdc.gov/view/cdc/126383). During 2022, acute flaccid paralysis (AFP) surveillance detected 127 paralytic cases, representing a 263% increase from 35 in 2020 and a 694% increase from 16 in 2021.

Since September 2020, Madagascar has experienced ongoing cVDPV1 transmission, with 13 cases detected during 2021 and 2022. Among three outbreaks active during 2021 (MAD-SUO-1, MAD-SUE-1, and MAD-ANO-1), the latest detection in the MAD-SUO-1 outbreak occurred in February 2021) (Supplementary Table, https://stacks.cdc.gov/view/cdc/126383) (*4*). An additional emergence (MAD-ANO-2) was confirmed in February 2022. DRC detected two cVDPV1 outbreaks in 2022 (RDC-TAN-1 in September and RDC-HLO-3 in November), totaling 91 cases by December and accounting for 72% of the global cVDPV1 cases in 2022. DRC also has concurrent cVDPV2 outbreaks.

In Mozambique, the first identified patient in the MOZ-NPL-2 emergence outbreak had paralysis onset in July 2020 (at that time an unclassified VDPV1 case); after the identification of additional genetically linked cases, an outbreak was confirmed in July 2022. Genomic sequence analysis indicated that the emergence had occurred approximately 4 years before the first detection, indicating substantial gaps in poliovirus surveillance (*7*). The MOZ-NPL-2 emergence spread to Malawi, where circulation was identified in September 2022 (*8*). Mozambique also has a concurrent wild poliovirus type 1 outbreak linked to Malawi (*9*). The latest detection of transmission of the Yemen outbreak (YEM-SAD-1 emergence) was in January 2021 (Supplementary Table, https://stacks.cdc.gov/view/cdc/126383).

## cVDPV2 Outbreaks

As of December 31, 2022, and since August 2016, a total of 154 cVDPV2 outbreaks from 82 cVDPV2 emergences have been reported in 48 countries. Seventeen (35%) countries reported their first post-switch cVDPV2 emergences and outbreaks in 2021 (eight) and 2022 (nine). Of the 82 emergences detected, 42 (51%) were active during 2021–2022, including nine (11%) new emergences identified in 2021 and five (6%) in 2022 (Table 1). Thirteen (16%) of all 82 emergences spread outside the country of first detection. The NIE-JIS-1 emergence, first detected in January 2018 in Nigeria, has spread to 18 other African countries; active transmission occurred in 13 of those countries during the reporting period. The NIE-ZAS-1 emergence, originally detected in Nigeria in July 2020, has been detected in an additional 12 countries since 2021. The YEM-TAI-1 emergence, first detected in Yemen in 2021, has spread into Egypt and Somalia, and the SOM-BAN-1 emergence group, first detected in Somalia in October 2017, continues to circulate only in that country (*4*).

Among the 154 cVDPV2 outbreaks that have occurred since the 2016 global synchronized switch from tOPV to bOPV for routine immunization, 76 (49%) were active across 42 countries during the reporting period (Figure). Forty-nine (65%) of the active outbreaks across 35 countries were first reported during 2021–2022 (Table 1). Even as cVDPV2 outbreaks have spread, the reported number of paralytic cases has declined from the peak in 2020: 587 paralytic cVDPV2 cases were reported in 2022 (as of February 14, 2023), representing a 14% decrease from 682 in 2021 and a 46% decrease from 1,082 cases in 2020. However, 2022 case counts could ultimately match or surpass 2021 counts as samples collected from the end of 2022 are processed. In 34 (45%) of the 76 active outbreaks, the latest detections occurred ≥13 months earlier, and transmission in those outbreaks is considered interrupted (Supplementary Table, https://stacks.cdc.gov/view/cdc/126383).

**FIGURE:**
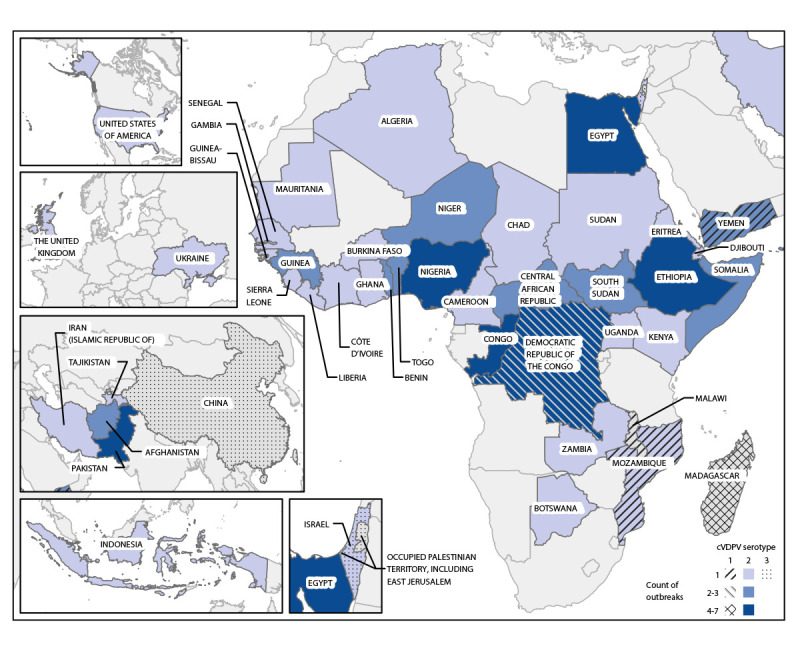
Circulating vaccine-derived poliovirus outbreaks (N = 88) — worldwide, January 2021–December 2022* **Abbreviation:** cVDPV = circulating vaccine-derived poliovirus. *Data current as of February 14, 2023.

Among the 49 new cVDPV2 outbreaks that occurred during the reporting period, 33 occurred in 23 countries in the WHO African Region (AFR), and 10 new outbreaks occurred in six countries of the WHO Eastern Mediterranean Region (EMR). Nine countries (Algeria, Botswana, Eritrea, The Gambia, Guinea-Bissau, Mauritania, Senegal, South Sudan, and Uganda) in AFR and two (Djibouti and Yemen) in EMR reported their first post-switch cVDPV2 outbreaks during this period. Among the 41 cVDPV2 outbreaks that were active at the end of the reporting period, 27 (66%) were in 19 AFR countries and nine (22%) were in five EMR countries (Table 1). In 2022, cVDPV2 cases from outbreaks in two countries (DRC [AFR] and Yemen [EMR]) represented 75% of all type 2 cases reported. The DRC reported 283 cases, an increase of 911% from the 28 cases reported in 2021, representing 48% of global cVDPV2 cases. Yemen reported an increase of 142%, from 66 cases in 2021 to 160 cases in 2022, accounting for 27% of global cVDPV2 cases in 2022.

The PAK-GB-1 emergence detected in Pakistan in 2019 spread to Tajikistan in 2020 and subsequently to Ukraine, with two cases identified during October–December 2021 (*4**,**6*). In 2022, genetically related VDPV2 detections (IUUC-2022) were reported in Israel, the United Kingdom, and the United States (New York) (*10*). One polio case and 12 environmental surveillance (ES) isolations were reported in the United States, five nonpatient isolations in the United Kingdom, and one nonpatient isolation in Israel. In Indonesia, a new cVDPV2 outbreak (INO-ACE-1) with one case was reported in November 2022. Genetic sequencing analysis suggested the emergence strain had been circulating undetected for approximately 3 years.

## cVDPV type 3 (cVDPV3) Outbreaks

Four cVDPV3 outbreaks from different emergences have occurred since 2016, two of which were active during the reporting period. One outbreak was in Israel (cVDPV3-ISR) during 2021–2022, with one paralytic case, and one in the Palestinian Territories (cVDPV3) in 2022, with 16 ES detections (Table 1).

## Outbreak Control

Of the 172 cVDPV outbreaks reported since 2016, 121 (70%) have been interrupted. A current critical measure of outbreak response performance for the Global Polio Eradication Initiative (GPEI) is the interruption of virus transmission in outbreaks (i.e., the latest detection) within 120 days of the outbreak notification date (*1*). As of February 14, 2023, 19 of 29 (66%) outbreaks confirmed in 2021 had no virus detected after 120 days, compared with 28 (62%) of 45 outbreaks in 2019 and 27 (54%) of 50 in 2020 (Table 2).

**TABLE 2:** Circulating vaccine-derived poliovirus outbreaks (N = 149) and timeliness of outbreak control, by serotype and year of confirmation — worldwide, August 2016–December 2022

cVDPV type	Year of outbreak confirmation, no. (%)						
	2016	2017	2018	2019	2020	2021	2022*
Type 1	—^†^	—	1	4	1	3	1
Type 2	2	4	7	41	49	24	8
Type 3	—	—	1	—	—	2	1
**Overall**	**2**	**4**	**9**	**45**	**50**	**29**	**10**
**Controlled within 120 days of outbreak confirmation (n = 85 [57%])**							
Type 1	—	—	0 (—)	4 (100)	0 (—)	1 (33)	0 (—)
Type 2	2 (100)	3 (75)	1 (14)	24 (59)	27 (55)	16 (67)	4 (50)
Type 3	—	—	0 (—)	—	—	2 (100)	1 (100)
**Overall**	**2 (100)**	**3 (75)**	**1 (11)**	**28 (62)**	**27 (54)**	**19 (66)**	**5 (50)**

**Abbreviation:** cVDPV = circulating vaccine derived poliovirus.

* Data as of February 14, 2023. To account for potential low-level transmission continuing after the outbreak response and delayed detection, cVDPV outbreaks were suppressed if <6 months had elapsed from the 120th day following outbreak confirmation (April 20, 2022).

^†^ Dashes indicate that no outbreaks were confirmed during that year.

## Discussion

GPEI’s 2022–2026 strategic plan includes the goal of stopping all cVDPV outbreaks by the end of 2023. Ongoing global cVDPV2 transmission and an increasing number of cVDPV1 outbreaks, with cocirculation of cVDPV1 and cVDPV2 in two countries, threaten the attainment of this target (*1*). Although the number of cVDPV2 cases and of new reported emergences have decreased during 2021 and 2022, two major challenges to reaching the target remain: 1) achieving high-quality surveillance that detects poliovirus in a timely manner, and 2) implementing fully effective outbreak control measures that prevent international spread. Wide gaps in poliovirus surveillance led to late detection of some countries’ outbreaks (e.g., MOZ-NPL-2), inferred by the extent of the genetic divergence of the initial isolates.

The number of paralytic cVDPV2 cases reported in 2022 represents a 46% decrease from the peak number in 2020 (*4**,**9*). During the initial months of the COVID-19 pandemic (March–June 2020), polio outbreak response SIAs were postponed. Most SIAs during the successive months of the reporting period were either delayed or of poor quality, resulting in the detection of breakthrough^¶^ cVDPV viruses in many outbreaks (*2**,**4*). The proportion of outbreaks controlled within 120 days has not substantially changed from that during previous years.

The decrease in number of new cVDPV2 emergences during this period is likely associated with the use of nOPV2 for outbreak response campaigns. Since the first cVDPV2 outbreak response using nOPV2 under the WHO Emergency Use Listing in March 2021 (as of March 2023), >590 million nOPV2 doses** have been administered in 24 countries (*5*). Whereas the number of cVDPV2 emergences has declined during the 2021–2022 COVID-19 pandemic and recovery period, international spread has not. During the last 2 years, 17 countries have experienced their first post-switch cVDPV2 outbreaks, reflecting poor outbreak control in the country of origin.

In 2022, the number of new cVDPV1 outbreaks increased substantially and primarily affected countries in sub-Saharan Africa. Routine immunization coverage, which was already low in many subnational areas of outbreak countries, decreased after the start of the COVID-19 pandemic, and the suspension of preventive bOPV SIAs has resulted in an environment with increased susceptibility to the emergence of cVDPV1 outbreaks (*6*). During 2022, in AFR countries, the national proportion of children who received their third dose of polio vaccine (Pol3) by age 1 year was 70%, compared with 74% in 2019; Pol3 coverage in EMR was 83% both years (*6*). Increasing routine immunization coverage will be critical for preventing paralysis and aiding in the interruption of global cVDPV1 transmission.

The findings in this report are subject to at least two limitations. First, delays in shipment and testing of poliovirus surveillance specimens by regional or international reference laboratories might have resulted in delays in detection of emergences and of additional cases during the second half of 2022. Second, surveillance gaps might have resulted in underestimates of poliovirus cases and the extent of transmission.

Countries responding to cVDPV outbreaks face multiple challenges in implementing effective outbreak responses, including delays in outbreak detection and receipt of vaccine, resulting in substantial transmission before implementation of response SIAs. Countries face competing public health priorities (e.g., outbreaks of measles, cholera, and Ebola virus disease), security challenges, and other national priorities with limited resources, which can negatively affect the overall quality and timeliness of outbreak response SIAs. Recent limitations of sufficient nOPV2 availability have hampered timely SIAs in response to cVPDV2 outbreaks. Thus, improving routine immunization coverage, especially at subnational levels, strengthening poliovirus surveillance, and conducting timely and high-quality outbreak response SIAs will be critical to interrupt cVDPV transmission in outbreaks and reach GPEI’s goal of no cVDPV isolations in 2024.

**Summary**

**What is already known about this topic?**

Circulating vaccine-derived polioviruses (cVDPVs) can emerge and cause paralysis in areas with low population immunity to polioviruses.

**What is added by this report?**

During January 2021–December 2022, 76 cVDPV type 2 outbreaks occurred in 42 countries. Since 2020, the numbers of paralytic cases and new emergences have declined following the introduction of a safer novel type 2 oral poliovirus vaccine for outbreak control. The number of cVDPV type 1 outbreaks increased during 2021–2022 as COVID-19 pandemic–associated global routine immunization coverage declined.

**What are the implications for public health practice?**

Improving routine immunization coverage, strengthening poliovirus surveillance, and conducting timely and high-quality supplementary immunization activity responses to cVDPV outbreaks in 2023 are necessary to stop cVDPV transmission.

## Footnotes

* By genomic sequence analysis of the region encoding capsid viral protein 1 (VP1), a poliovirus with >1% divergence from the parent Sabin strain for serotypes 1 and 3, or >0.6% for serotype 2 is classified as a VDPV. Evidence of circulation (i.e., a cVDPV outbreak) occurs when two or more independent detections of genetically linked VDPVs are identified through AFP surveillance, environmental surveillance, or from healthy community members.

† In April 2016, all OPV-using countries withdrew tOPV from routine immunization activities and switched to bOPV. Each OPV serotype induced protection against paralysis and poliovirus transmission. Monovalent OPV type 2 (mOPV2), tOPV (when there is cocirculation), and novel OPV type 2 (nOPV2) are reserved for use in cVDPV2 outbreak response SIA. At least 1 dose of injectable inactivated poliovirus vaccine (IPV) is included in routine immunization. IPV induces production of antibodies that protect a person against paralysis from all three poliovirus serotypes, but it does not stop poliovirus transmission.

§ VDPV emergences are defined by shared genetic changes in the VP1 capsid region from the parental OPV strain, not genetically related to previous VDPV detections. If an emergence group poliovirus is imported into other countries identified by one or more detections, those are considered additional outbreaks.

¶ Breakthrough transmission is defined as detection of a poliovirus (wild poliovirus or cVDPV) in samples from a patient with AFP, a healthy child, or environmental sampling sites with the date of onset of paralysis (for AFP cases) or the date of sample collection (for healthy children or environmental samples) >21 days after the first day of the last SIA in an area where at least two SIAs have been implemented.

** On March 16, 2023, GPEI released a statement on cVDPV2 detections from Burundi and DRC that have been linked with nOPV2 use. https://polioeradication.org/news-post/gpei-statement-on-cvdpv2-detections-in-burundi-and-democratic-republic-of-the-congo/
